# A framework for quantifying net benefits of alternative prognostic models‡

**DOI:** 10.1002/sim.4362

**Published:** 2011-09-09

**Authors:** Eleni Rapsomaniki, Ian R White, Angela M Wood, Simon G Thompson, Emerging Risk Factors Collaboration

**Affiliations:** a Department of Public Health and Primary Care, University of CambridgeCambridge, UK; b MRC Biostatistics UnitCambridge, UK; c ERFC Coordinating Centre, Department of Public Health and Primary Care, University of Cambridge, Strangeways Research LaboratoryCambridge CB1 8RN, UK

**Keywords:** net benefit, cost-effectiveness, cardiovascular disease, meta-analysis, competing risks, screening strategies

## Abstract

New prognostic models are traditionally evaluated using measures of discrimination and risk reclassification, but these do not take full account of the clinical and health economic context. We propose a framework for comparing prognostic models by quantifying the public health impact (net benefit) of the treatment decisions they support, assuming a set of predetermined clinical treatment guidelines. The change in net benefit is more clinically interpretable than changes in traditional measures and can be used in full health economic evaluations of prognostic models used for screening and allocating risk reduction interventions. We extend previous work in this area by quantifying net benefits in life years, thus linking prognostic performance to health economic measures; by taking full account of the occurrence of events over time; and by considering estimation and cross-validation in a multiple-study setting. The method is illustrated in the context of cardiovascular disease risk prediction using an individual participant data meta-analysis. We estimate the number of cardiovascular-disease-free life years gained when statin treatment is allocated based on a risk prediction model with five established risk factors instead of a model with just age, gender and region. We explore methodological issues associated with the multistudy design and show that cost-effectiveness comparisons based on the proposed methodology are robust against a range of modelling assumptions, including adjusting for competing risks. Copyright © 2011 John Wiley & Sons, Ltd.

## 1. Introduction

Accurate estimation of patient prognosis is important in clinical practice. For example, a 10-year predicted risk of cardiovascular disease events in the disease-free population, assessed on the basis of vascular risk factors including age, sex, smoking status, history of diabetes and levels of blood pressure and blood cholesterol, is commonly used to determine eligibility for preventive treatments such as cholesterol-lowering statin medications [1]. When new risk factors are proposed for inclusion in such prognostic models, they have traditionally been evaluated by their statistical significance or by the change in summary measures of predictive ability such as the C-index [2]. However, these approaches ignore the clinical context [3].

Recent work has focussed on developing measures of prognostic ability that are linked to clinical outcomes. Reclassification tables stratify individuals into a small number of risk groups and compare their classification in models with and without the new risk factor [4]. These comparisons can be quantified by the net reclassification improvement (NRI), the integrated discrimination improvement [5] and their extensions that take survival probabilities and censoring into account [6, 7]. A major advantage of these measures is that they are simple to implement and easy to communicate to clinicians. Despite using clinically relevant categories, however, their use and interpretation requires caution [8]. One issue is that true and false positives are implicitly weighted by the event prevalence in the data, leading to possibly misleading conclusions when the implied weights are not clinically meaningful [9]. Another difficulty arises when a study contains insufficient information (e.g. number of events, sample size, follow-up time) for the chosen risk categories. These can be tackled using a category-free formulation of the NRI, which builds in cost considerations by explicit weighting of different outcomes [10].

Approaches based on decision theory may have more specific relevance to clinical applications. These evaluate risk models in terms of the treatment decisions they support, expressing costs and benefits on the same scale using utilities [11]. Decision curve analysis (DCA) is a simplification of the decision theory approach that does not require utilities to be known [12]. Instead, individuals are assumed to vary in the threshold of risk at which they would favour a clinical intervention, where such thresholds reflect their personal relative utilities of a true positive (being treated when an event would occur) and a false positive (being treated when an event would not occur). The net benefit is computed in units of true positives and graphed against the risk threshold, with the possibility to allow for censored time-to-event outcomes [13]. DCA is equal to the weighted NRI in the two-category case when the threshold used defines equivalent weights, and also equal to the original NRI when utilities are based on event rates [10]. An extension to DCA is the relative utility curve, defined as the net benefit divided by the net benefit of a perfect model [14].

In this paper, we introduce new methods to assess differences in predictive performance quantitatively, producing a summary statistic – the net benefit – which is more interpretable than changes in traditional measures, and which can be meaningfully compared with data collection and other costs either informally or in a full economic evaluation. The proposed framework has three key features. First, we estimate the net benefit in units of event-free life years (EFLYs), which is appropriate for prognostic models, whereas considering binary outcomes is appropriate for diagnosis purposes. Second, we take full account of the occurrence of events over time, adjusting for censoring by using the Kaplan–Meier methods. Third, we extend our methods to the meta-analysis setting based on multiple prospective studies. Our methods follow DCA in assuming that the risk threshold is informative about utilities, but we focus on a situation where the risk threshold does not vary between individuals. The framework is developed for prognostic applications where the aim is to identify high-risk individuals who would benefit from risk reduction interventions administered over time. In this respect the scope differs from DCA, which focuses principally on diagnosis.

We develop the methods in the context of prevention of cardiovascular disease (CVD), although they are also applicable for other diseases. Cardiovascular disease risk prediction is routinely used in clinical practice to determine eligibility for preventive treatments [16]. For individuals whose 10-year predicted CVD risk exceeds a given threshold, guidelines advocate prescribing statins, a relatively low-cost medication with long established clinical effectiveness and few reported side-effects [15]; in the UK this threshold is currently 20% [16]. The choice of model to estimate CVD risk, and in particular the inclusion of new risk factors, is still a subject of intense research. It is important that new models and new screening options be with are properly evaluated by considering both health benefits and costs to inform public health policy. In this paper, we demonstrate the methodology by comparing a standard model with a simplified model, but the methods can also be used to evaluate models including new risk factors.

This paper is arranged as follows. In Section 2 we outline our data and the problem of CVD prevention. In Section 3 we set out our approach in generic terms, stating assumptions, defining the quantities of interest, and explaining how to estimate them in a single study. In Section 4 we describe the methods used in our analysis, extending to multiple studies, allowing for between study heterogeneity and tackling obstacles such as limited follow-up in some studies. In Section 5 we extend the methodology to adjust for competing risks and increased with increase precision in cost-effectiveness comparisons. Results are described in Section 6. In Section 7 we discuss our choice of methodology and the issues we tried to address in our sensitivity analyses, highlight the limitations of our approach and suggest ways to overcome them. We conclude with practical considerations and extensions needed to use this method in an applied health economic analysis, where a range of other costs and benefits from external sources would also be incorporated in the final estimates.

## 2. Motivating example

### 2.1. Data

The Emerging Risk Factors Collaboration (ERFC) has collated and harmonised individual participant data from population-based prospective studies of CVD [17]. In October 2009 the data set comprised 1.2 million individuals in 117 studies with an average of 12.5 years follow-up. We use these data to model time to first fatal/nonfatal CVD event, under which we include coronary heart disease and stroke. Our main analysis was restricted to prospective cohorts that record both fatal and nonfatal outcomes and provide information on history of diabetes, total cholesterol, blood pressure and smoking status at the baseline survey. Individual participant data were further restricted to participants aged at least 40 years at baseline with the above risk factors recorded, no history of diabetes or CVD recorded at baseline and not under statin treatment (when this information is provided). Thus, prognostic models were based on 12,058 CVD events recorded among 171,175 individuals from 53 studies. Studies were grouped into four geographic regions: USA and Canada, Northern Europe, Southern Europe and Japan. The data are summarized in Table A1.

### 2.2. Prognostic models considered

We compared CVD risk models for which the difference in prognostic performance is undisputed, in order to explore whether the new methodology leads to the expected conclusions. Model M_1_ includes gender, region, age and year of birth, while model M_2_ additionally includes three established CVD risk factors: systolic blood pressure, total cholesterol and smoking status (current versus other). Year of birth was included to adjust for possible birth cohort effects. The aim of our analysis is to estimate how much more cost-effective are treatment decisions based on model M_2_ compared to model M_1_ when the models are used to identify and treat a subgroup of high-risk individuals. We account for differences between the models both in CVD events prevented over 10 years and in numbers treated. Deaths from non-CVD causes are initially considered as censored observations, but we address the issue of competing risks in Section 5.

M_1_ and M_2_ are Cox proportional hazards models stratified by gender and region using age at risk as the timescale. Thus, for individual *i* of gender *g* from region *r*, the hazard at age *t* years is


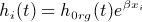
(1)

where *x*_*i*_ is the vector of covariates specified by a given prognostic model for individual *i*, *β* is a vector of corresponding regression coefficients and *h*_0*rg*_(*t*) is the baseline hazard at age *t* for individuals from region *r* and gender *g*. Although each region comprises several studies, we do not use study-specific baseline hazards, because the individuals to whom prediction models are applied in clinical practice belong to a region and not to a study; stratifying by cohort could give unrealistically good calibration and discrimination. However, by stratifying by region we allow the possibility of confounding by study; we address this issue in Section 4.1. These models use age as timescale [18]; we examined the effect of using time on study as the timescale in later sensitivity analyses.

We found that coefficients for blood pressure, total cholesterol and smoking status are significantly greater at younger ages. Hence, model M_2_ was further refined by allowing the coefficients for these covariates to take different values in individuals aged under and over 55 at baseline, the median baseline age in our data. For simplicity we did not consider further refinement, for example, using more than two age groups or adding age as a continuous term in interactions. A simplified version of M_2_ that used the same coefficients at all ages is examined in sensitivity analysis I.

## 3. Estimating net benefit from a prognostic model

In this section we describe how we estimate the net benefit associated with a screening model, which is a risk model used to identify individual risk in a population, using data from a single study. This section assumes a background understanding of basic survival analysis techniques at the level covered in any good introductory textbook in this field (e.g. [19]).

### 3.1. Approach

We assume that a prognostic model is used to compute a cumulative risk *r*_*i*_(*T*) for each individual *i* up to time *T* years, and that there is a treatment threshold *c* so that ‘high-risk’ individuals with *r*_*i*_(*T*) *≥ c* receive an intervention that multiplies their hazard of disease by hazard ratio *θ* < 1. The benefit of this package (prognostic model, risk calculation, selection and treatment) is the reduction in disease events, and the cost is proportional to the amount of treatment received.

To evaluate the benefit and cost of the package, we make the following assumptions. Benefit and cost are calculated over a time period *T*, the ‘time horizon’. Individuals are not re-assessed before time *T*. If allocated to treatment, individuals will be 100% compliant with treatment up to time *T*. Screening costs are not included. We ignore any impact of treatment on lifetime after (nonfatal) disease events. We assume proportional hazards for risk factors and treatment effects. Finally, the hazard ratio for treatment, *θ*, is the same across different subgroups. We discuss how to relax these assumptions in Section 7.

### 3.2. Benefits and costs in terms of event-free life years

The following quantities are defined within the group of ‘high-risk’ individuals. Suppose *S*(*u*) is the probability of survival to time *u* under no treatment. Assume that a treatment with hazard ratio *θ* is administered until death. The cumulative risk to time *u* is then reduced by *S*(*u*)^*θ*^ −*S*(*u*) and the number of EFLYs gained as a result of treatment within the time horizon is 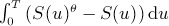. If *μ* is the monetary worth of one EFLY, then the monetary benefit *B*^*‵*^(*T*) to time *T* per person treated is


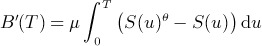
(2)

Similarly, if *ν* is the monetary cost of treatment per person per year, then the average cost of treatment at time *u* is *νS*(*u*)^*θ*^, so the treatment cost per person over the time horizon is


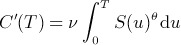
(3)

The quantities above are defined in monetary units. To express them in EFLY units we divide by *μ* and set *k* = *ν*/*μ*, the cost of treatment per year relative to the value of one EFLY, giving


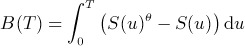
(4)


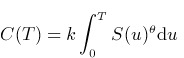
(5)

We estimate *B*(*T*) and *C*(*T*) by applying a trapezium rule approximation


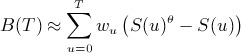
(6)


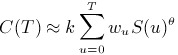
(7)

where the sums go in steps of time *d*, *w*_0_ = *w*_*T*_ = *d*/2 and *w*_1_ = … = *w*_*T*−*d*_ = *d*.

Let *P* be the proportion treated among those screened. Then the benefit to time *T* per person screened (in EFLY units) is *P* ×*B*(*T*) and the net benefit is:


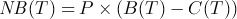
(8)

Finally, we use this procedure to compare two models M_1_ and M_2_, adding a (final) suffix *m* = 1 or 2 to the notation where necessary. Then the difference *DNB*(*T*) = *NB*_2_(*T*) −*NB*_1_(*T*) represents the number of EFLYs gained up to time *T* if M_2_ is used instead of M_1_ for screening. A numerical example demonstrating the steps for estimating *DNB*(*T*) in a hypothetical cohort is shown in Table A2.

### 3.3. Estimation of the ‘optimal’ cut-off and k forrational treatment

From Equation (4), it follows that individuals with higher risk receive a larger treatment benefit. We assume that the value of *c* specified by existing guidelines is a rational treatment cutpoint, in the sense that benefits equal costs when risk is *c*.

We now relate *k* to *c*, *T* and *θ* using a working assumption of exponential survival. For an individual with risk *r*_*i*_(*T*) = *c*, we approximate *S*(*t*) ≍ *e*^−*λt*^ where *λ* = −(log(1 −*c*)/*T*). Evaluating the integrals in Equations (2) and (3) shows that treating this individual yields benefit


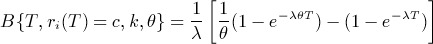
(9)

and cost


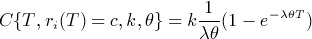
(10)

Setting (9) and (10) equal gives


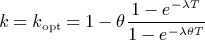
(11)

In practice, clinical guidelines on treatment thresholds are not based on balancing costs and benefits alone as we describe here; a wealth of empirical evidence such as the burden of disease, the availability of alternative treatments and uncertainty around estimates contribute to the chosen thresholds [20]. However, comparing the cost-effectiveness between models using values for *k*, *c* and *θ* that violate condition (9) can lead to unexpected and misleading conclusions. For example, suppose that the costs associated with an effective treatment are negligible, so that all treatment is beneficial even in low-risk individuals. In this situation it is reasonable to ‘treat all’ (set *c* = 0). If nevertheless a high *c* is chosen, a miscalibrated model that consistently over-predicts risk would lead to larger net benefit than a well calibrated model that more appropriately assigns people to lower risk categories, simply because more individuals would be treated. The conclusion that the miscalibrated model is more cost-effective would be misleading in the sense that it is confounded by the irrational choice of treatment threshold.

Our main analyses are therefore ‘optimal-cutpoint’ analyses that assume that *k* = *k*_opt_. Where we examine the effect of varying *c* or *θ*, we recalculate *k* from Equation (9).

### 3.4. Estimation of net benefit

The method is easily implemented using a single prospective study data set with follow-up beyond time *T*, excluding individuals recorded to be already under CVD-risk reducing treatments; estimation methods in the more complex multiple study setting are discussed in the next section.

In the risk estimation stage, the Cox model (1) is fitted to the data, and the fitted model (including estimated baseline hazard) is used to estimate predicted risks *r*_*i*_(*T*) for each individual *i*. This is done separately for models M_1_ and M_2_.

Next, given *θ* and the ‘optimal-cutpoint’ *c*, we determine *k* using Equation (9). In our main analysis we assume that *c* is optimal for treating under either model.

In the evaluation stage, we find the ‘high-risk’ groups with *r*_*im*_(*T*) > *c*_*m*_ and estimate their prevalence *P*_*m*_ and survivor functions *S*_*m*_(*t*) using Kaplan–Meier or other methods. Equations (5)–(6) now give *NB*_*m*_(*T*).

We extend this estimation procedure using 10-fold cross-validation to avoid ‘optimism’ in model evaluation [21]. In the risk estimation stage, the data are randomly divided into 10 subgroups. The risks *r*_*i*_(*T*) for individuals in subgroup *q* are estimated by fitting the Cox model (1) to all subgroups except subgroup *q*. Repeating this for each subgroup *q* = 1, …,10 yields predicted risks *r*_*i*_(*T*) for all individuals. Finally, we estimate standard errors by repeating the whole estimation procedure on a suitable number (200) of bootstrap samples.

We examined the effect of cross-validation on our estimates by omitting it in sensitivity analysis II, that is, fitting and applying the models to the same data. To explore the external validity of the models more stringently than using random split, in sensitivity analysis III we performed leave-one-study-out cross-validation, whereby risk in each study is computed using parameters estimated from the remaining studies.

### 3.5. Fixed-budget method

As an alternative to the ‘optimal-cutpoint’ analysis, we may view treatment allocation as controlled by a budget-holder who specifies the number of people that can be treated before the budget is exhausted. If *c*_1_ and *c*_2_ denote the cutpoints to use with M_1_ and M_2_, respectively, this implies that *c*_1_ ≠ *c*_2_. Hence, if we assume that the budget is sufficient to treat as many individuals as those whose risk to *T* under M_1_ (*r*_*i*1_(*T*)) equals at least *c*_1_, then to treat the same number under M_2_ we find the threshold *c*_2_ that gives *P*(*r*_2_(*T*) *≥ c*_2_) = *P*(*r*_1_(*T*) *≥ c*_1_) (where the probabilities are over the distribution of covariates *x*). The remaining steps to estimate net benefit proceed as described above, but costs are necessarily very similar for the two models, so the final estimated difference in net benefit does not depend on the value chosen for *k*.

## 4. Estimation methods in multiple studies

In this section, we describe how we estimate the quantities described in Section 3 in a multiple study setting such as the ERFC data. As in the single setting discussed above, the method involves two stages: risk estimation (finding *r*_*i*_(*T*) for each model) and model evaluation (finding *DNB*). Sampling for cross-validation and bootstrapping is stratified by study.

### 4.1. Risk estimation step

The risk model (1) cannot be directly estimated by a Cox model stratified by region, because this would introduce confounding by study. Instead, we adopted a two-step approach. We first fitted the Cox regression model 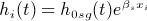 to each study *s*, where *β*_*s*_ is a study-specific vector of coefficients, *x*_*i*_ is the vector of covariate values for individual *i* and *h*_0*sg*_(*t*) is the study-specific and gender-specific baseline hazard at age *t*. We then combined each element in *β*_*s*_ using univariate random-effects meta-analysis (REMA) to obtain the vector of combined coefficients 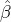. As a sensitivity analysis, we also combined coefficients using multivariate REMA [22] and confirmed that the two approaches result in a similar set of coefficients (results not shown).

To estimate region-specific and gender-specific Nelson–Aalen baseline hazards *h*_0*rg*_(*t*), we fitted a Cox model with an offset 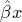 to fix the effect of covariates to their pooled estimates 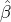:


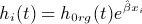
(12)

For individual *i*, the cumulative risk from age *t*_0*i*_ at study entry to *t*_0*i*_ + *T* was estimated as


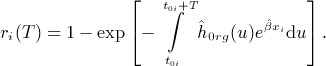
(13)

These steps were combined with the 10-fold cross-validation approach described in Section 3.

We explored how estimates were affected by modelling hazards parametrically, by study or by region, and with age or time-on-study as the timescale. In sensitivity analysis IV, baseline hazards were estimated parametrically, using a region-specific and gender-specific Weibull model. In sensitivity analyses V and VI, Equation (10) was fitted per study and gender (instead of per region and gender), with baseline hazards estimated by Nelson–Aalen (in V) or Weibull modelling (in VI). Finally, in models stratified by study and gender we investigated the effect of using time-on-study as the timescale, again with Nelson–Aalen baseline hazards (sensitivity analysis VII) or Weibull modelling (sensitivity analysis VIII).

### 4.2. Evaluation step

In the evaluation stage, we compute *P*, *B*(*T*) and *C*(*T*) using the individuals with *r*_*i*_(*T*) *≥ c*. Although the studies are heterogeneous, there is likely to be much less heterogeneity between studies among this treated group, most of whom have risk close to *c*. For our main analysis we therefore obtain a simple Kaplan–Meier estimate for survival in the treated *S*(*u*), treating this group as a single homogeneous dataset as in Section 3.

For sensitivity analyses, we explored alternative approaches for evaluating observed risk and combining study-specific estimates that more formally account for study heterogeneity. In sensitivity analysis IX, *S*(*u*) was computed as the mean of study-specific Kaplan–Meier 10-year estimates (extrapolated to 10 years where necessary as described in Section 4.3), weighting each study by its relative size within the treated group. A similar approach was used in sensitivity analysis X, but here estimates were combined at a later stage, after computing *NB* and *DNB* in each study. Study weights in this case were based on the total study size, so the *DNB* estimate was


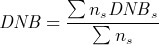
(14)

where *n*_*s*_ is the sample size of study *s* and *DNB*_*s*_ is the corresponding difference in the net benefit. Weighting the *DNB*_*s*_ by their inverse variances was not appropriate, because some low-risk studies had zero or few ‘high-risk’ subjects and hence had a variance that was zero or very small, leading to very high weights and distorted combined estimates.

### 4.3. Handling studies with follow-up < *T*years

The risk estimation and evaluation steps use *T*-year risks estimated in different subgroups – by region (for risk predictions), across all regions (in the evaluation step), or within each study (in some sensitivity analyses). Some of these subgroups had insufficient follow-up or too few events towards the start and/or end of the survival curve to provide reliable survival estimates. To overcome this problem, survival to *T* years was extrapolated assuming a Weibull distribution.

With age as timescale, extrapolation was necessary for individuals whose age after *T* years was above the maximum observed age with events. Extrapolation was also desirable near the left-most and right-most extremes of the survival curve, where information is sparse. Hence, we used extrapolated estimates for time points below *t*_low_ and above *t*_upp_, defined as the 5^th^ and 95^th^ centiles of the observed failure ages.

With time-on-study as timescale, extrapolation was necessary for subgroups with maximum follow-up < *T* years. In addition, extrapolation may also be desirable where the survival curve is based on small numbers; this was implemented in sensitivity analysis XI as an alternative way to estimate survival in the evaluation step. For this timescale, we extrapolated beyond time *t*_upp_ defined as the median censoring time (Table A1) in the subgroup and computed using ‘reverse Kaplan–Meier’ [23], whereby censoring is indicated by 1 and event by 0. To ensure that extrapolation affects the two models equally, the smaller *t*_upp_ and the larger *t*_low_ of the two models were used for both. Extrapolated survival to *t* was


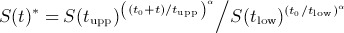
(15)

where, with age as the timescale, *t*_0_ corresponds to baseline age; with time-on-study as the timescale, *t*_0_ = 0 and (13) simplifies to 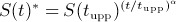. Equation (13) was used to extrapolate both predicted risk and (in the evaluation stage) Kaplan–Meier estimates. In the risk prediction stage, the shape parameter *α* was estimated from region-specific Weibull models adjusting for the pooled linear predictors. In the evaluation stage, *α* was estimated from a Weibull model fitted without covariates to the treated group from each model across all regions using time-on-study as the timescale.

## 5. Methodological extensions

### 5.1. Adjusting for competing risks

So far we have treated competing (non-CVD) causes of death as censored. Estimated risks therefore include CVD events that might have been observed if non-CVD deaths did not occur. By allowing for competing risks, we instead estimate the true risk of a CVD event actually occurring to an individual, which seems a more sensible basis for a treatment decision [24]. Adjusting for competing risks is similarly important in the evaluation stage.

In the risk estimation stage, a competing risks version of the Cox model was used in each study [25]. The cause-specific hazard for individual *i* from competing cause *k* was 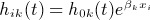, where *h*_0*k*_(*t*) is the Nelson–Aalen cause-specific baseline hazard at age *t* and *β*_*k*_ is a vector of cause-specific coefficients. Estimated cause-specific baseline hazards were extrapolated to 10 years where necessary, as described in Section 4.3. Letting cause 1 be CVD and cause 2 be non-CVD deaths, the estimated cumulative incidence *r*^CR^(*T*) of CVD from age *t*_0_ to age *t*_0_ + *T* given covariates *x* was


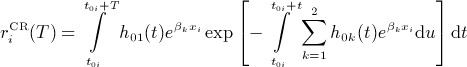
(16)

We examined two ways to specify the treatment threshold with cumulative risks. The ‘crude risk cutpoint’ (sensitivity analysis XII) uses the same treatment threshold for 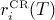 as for *r*_*i*_(*T*). Because 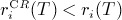, fewer individuals are treated under a competing risks model. The alternative ‘cumulative risk cutpoint’ (sensitivity analysis XIII) reduces the cut-off for each model so that the same number of individuals are treated as under crude risks, that is 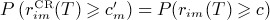 for *m* = 1,2.

To adjust for competing risks in the evaluation stage, we assumed that treatment has no effect on the competing risks. To estimate events averted over 10 years in the treated, we used the competing risks model without covariates and with time-on-study as the timescale, so that *t*_0_ = 0. Letting *h*_*k*_(*u*) = −log (*S*_*k*_(*u*)) where *S*_*k*_(*u*) is the Kaplan–Meier estimate to time *u* for cause *k*, we can replace *S*(*u*) and *S*(*u*)^*θ*^ in Section 3 by 1 −*I*^CR^(*u*) and 1 −*I*^CR^(*u*; *θ*), where


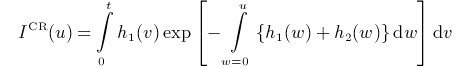
(17)


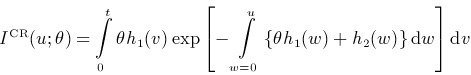
(18)

### 5.2. Gaining precision

Because the number of ‘high-risk’ individuals can be small, Kaplan–Meier estimation of 10-year risk in the treated can be imprecise. Two approaches were considered to increase precision in the evaluation step. The first replaces observed risks with risks predicted using a rich and well calibrated model M^*^, as suggested in [14]. We apply this in sensitivity analysis XIV, taking M^*^ to be M_2_ stratified by study and gender (see Table A3, sensitivity analysis VII).

The other approach is based on ‘smoothing’ around the risk threshold. All individuals with predicted risk above *c* contribute equally to the evaluation stage, but greater precision may be gained by exploiting the likely similarities between individuals with predicted risk just below and just above *c*. We do this by modelling treatment probabilistically rather than deterministically. Specifically, we assume that individuals with *r*_*i*_(*T*) = *c* have 50% chance of treatment, and that chance of treatment varies linearly with *r*_*i*_(*T*), from 0 at *r*_*i*_(*T*) = *c*−*δ* to 1 at *r*_*i*_(*T*) = *c*+ *δ*. This probability of treatment was used as a weight throughout the evaluation stage. We demonstrate this approach in sensitivity analysis XV, where we use *δ* = 5*%*.

## 6. Results

The values assumed for *T*, *c*, *θ* and *k* are shown in Table I. Our choice for *T* and *c* is based on current clinical guidelines for CVD prevention [1] while *θ* is based on meta-analysis of statin trials [15]. Based on these values we estimated *k* using Equation (9). To aid interpretation of *k*, if the monetary value of 1 EFLY is £20000 (as proposed by NICE [Bibr b26]) and the cost of treating one person per year is £426 then the ratio of treatment cost to EFLY value is *k* = *£*426/*£*20000 = 2.13*%*. Note that the treatment cost of £426 is estimated directly from Equation (9) to justify the threshold of 20% (for *T* = 10 years) if 1 EFLY can be exchanged with £20000 and *θ* = 0.8.

**Table I:** Reference values used for treatment efficacy, treatment cost relative to EFLY cost and the risk threshold above which treatment is assumed. The values shown for the main analysis are incorporated in all estimates of cost-effectiveness unless stated otherwise

Quantity	Symbol	Value in main analysis	Values in sensitivity analysis
Time horizon	*T*	10 years	
Risk threshold for treatment	*c*	20% at 10 years	10%, 30%
Treatment hazard ratio	*θ*	0.8	0.7, 0.9
Treatment cost relative to EFLY cost^a^	*k* = ν/μ	2.13%	1%, 3.3%

a Value of *k* is computed from *c* and θ using Equation (9).

ν, cost of treatment per person per year; μ, monetary value of EFLY.

### 6.1. Distribution of 10-year risk

The observed 10-year CVD risk across all the data is 6.7%. Combined coefficients for each model are shown in Table II. Pooled proportional hazard ratios for smoking, total cholesterol and blood pressure were significantly higher in younger individuals (aged < 55 years at baseline).

**Table II:** Coefficients for models M_1_ and M_2_ estimated from study-specific Cox models stratified by gender and combined by univariate random-effectsmeta-analysis

Covariate	Hazard ratio	(95% CI)
*Model M*_1_		
Year of birth	0.994	(0.978 to 1.010)
*Model M*_2_		
Year of birth	1.009	(0.992 to 1.027)
Baseline age ≥ 55	1.141	(0.981 to 1.329)
Current smoker : baseline age < 55	2.182	(1.959 to 2.431)
Current smoker : baseline age ≥ 55	1.711	(1.562 to 1.875)
Total cholesterol : baseline age < 55	1.288	(1.241 to 1.337)
Total cholesterol : baseline age ≥ 55	1.118	(1.086 to 1.150)
Systolic blood pressure : baseline age < 55	1.021	(1.018 to 1.023)
Systolic blood pressure : baseline age ≥ 55	1.014	(1.012 to 1.015)

*Note*: Total cholesterol in mmol/L, systolic blood pressure in mm Hg.

**Figure 1:**
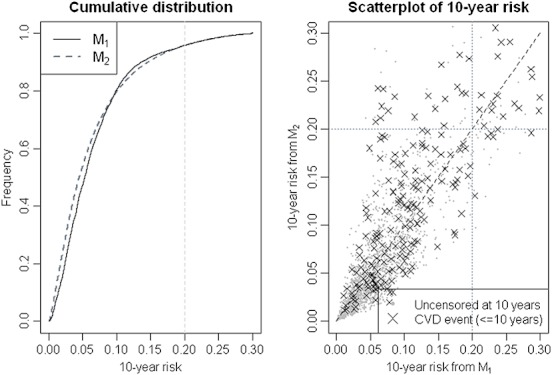
LEFT: the cumulative distribution of the 10-year CVD risk across all the data used in the main analysis as predicted by each model. RIGHT: Scatter plot of predicted 10-year risk (only a randomly selected 5% of the data is plotted). The data points highlighted in black correspond to individuals who experienced a CVD events within 10 years of follow-up, grey points indicate all others (individuals with no events before 10 years or censored). The dashed vertical and horizontal lines point to the 20% risk threshold. The dashed diagonal corresponds to the theoretical line of perfect correlation.

In Figure 1 we compare the cumulative distribution of 10-year risks predicted by each model. Model M_2_ predicts slightly fewer individuals in the 5% to 15% risk range compared to M_1_ (left panel of Figure 1) while the scatter-plot (right panel of Figure 1) shows a substantial difference between M_1_ and M_2_ risks. However, neither model could identify the majority of the individuals who experienced a CVD event within 10 years (black points) as high-risk. With both models, over 94% of individuals are predicted to be below the 20% risk threshold.

### 6.2. Concordance and calibration

Harrell's C-index 1996 measures the ability of a model to discriminate between low and high-risk individuals. A pair of risk predictions is concordant if a higher risk is predicted for the individual who experienced an event first. We computed region- and gender-specific C-indices by ranking patients according to their 10-year predicted risk and comparing pairwise concordance against their observed outcomes. The mean C-index across all the data (weighted by the number of events per region and gender) was 0.686 for M_1_ and 0.736 for M_2_, meaning that 5% more pairs have 10-year risk predictions concordant with their outcomes based on model M_2_ compared to M_1_.

**Figure 2:**
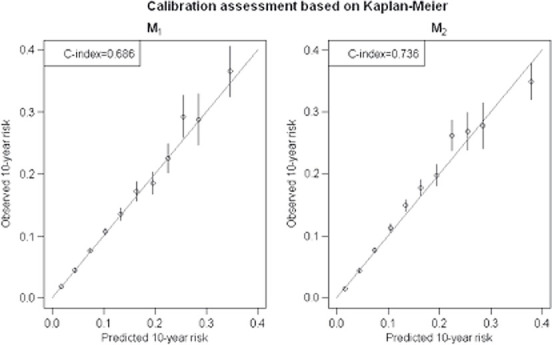
Calibration plot with 95% CIs for observed (1-KM) against predicted risk (mean risk within each risk group). Risk groups are model-specific and increase by 3% from 0 to < 30% (the last group is ≥30%). Estimation of the C-index is based on comparing predicted 10-year risk with observed outcomes between all comparable pairs irrespective of study origin.

Calibration assesses the correspondence between observed and predicted risk categories. We assessed calibration visually by splitting the data into model-specific risk groups and plotting the observed (Kaplan–Meier) risk for each group against its mean predicted risk (Figure 2). A reasonably good calibration was obtained at least up to 20% observed risks for either model. Because the 20% threshold is based on typical treatment decisions in medical practice in the UK [16], imperfect calibration at higher risks is expected to have a relatively low impact on the net benefit (individuals affected are still being treated). Hence, in terms of their calibration, both these models are useful for identifying which individuals will receive benefit if treated.

### 6.3. Net benefit (all data)

Estimates of net benefit and intermediate quantities involved in its computation are shown in Table III. With the ‘optimal cutpoint’ approach of Section 3, the ‘high-risk’ group identified by M_2_ is larger than that identified by M_1_. In addition, observed 10-year risk is higher in individuals identified as ‘high-risk’ by M_2_ compared to those identified by M_1_, resulting in larger treatment benefit and slightly lower cost per person treated. Putting these together, both models result in a net gain in EFLYs (positive NB), but M_2_ has a larger gain than M_1_.

**Table III:** Estimated quantities used to compute net benefit for models M_1_ and M_2_. Standard errors (SE) are based on 200 bootstraps

Quantity	‘Optimal cutpoint’			‘Fixed budget’	
		
	M_1_	M_2_	*M*_*2*_– *M*_*1*_*(SE)*	M_2_	*M*_*2*_– *M*_*1*_ *(SE)*
Treatment threshold (*c*)	20%	20%	*fixed by method*	20.96%	*0.96% (0.23%)*
Number treated per 1000 screened (*P* ×1000)	51.6	58.3	*6.71 (1.58)*	51.6	*fixed by method*
10-year risk in treated [1 − *S*(*T*)]	26.5%	28.2%	*1.7% (0.7%)*	29.1%	*2.6% (0.7%)*
Benefit per treated [ *B*(*T*)]	0.223	0.243	*0.020 (0.004)*	0.249	*0.026 (0.004)*
Cost per treated [ *C*(*T*)]	0.192	0.190	*–0.002 (0.0002)*	0.190	*–0.003(0.0003)*
*Estimates (in EFLYs) per 1000 screened*					
Benefit [ *P* × *B*(*T*) ×1000]	11.51	14.14	*2.64 (0.37)*	12.83	*1.32 (0.24)*
Cost [ *P* × *C*(*T*) ×1000 ]	9.92	11.10	*1.17(0.29)*	9.78	*–0.14 (0.02)*
Net Benefit [ *P* × (*B*(*T*) − *C*(*T*)) × 1000]	**1.58**	**3.04**	***1.46 (0.26)***	**3.04**	***1.46 (0.26)***

Estimated differences in net benefit based on the ‘fixed budget’ scenario are very similar, despite the differences in the number treated under M_2_. The similarity in these estimates comes about through the balance of two opposing features. Thus, under a fixed-budget treatment is allocated more efficiently, with cost saved by treating fewer individuals with the highest risk. In contrast, the optimum cut-off scenario incurs a higher cost by treating more individuals but also generates a higher overall benefit by spreading treatment across a larger number of individuals whose observed risk remains within the cost-effective area.

Our main result suggests that we will save 1.46 (SE 0.26) more EFLYs per 1000 people aged over 40 if we screen them using M_2_ instead of M_1_.

### 6.4. Net benefit by *M*_1_ riskgroup

**Figure 3:**
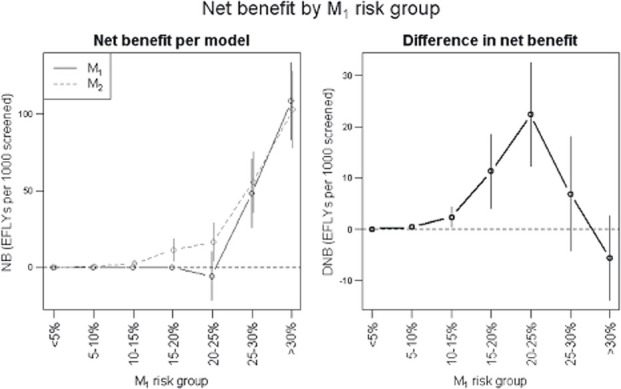
Comparisons of net benefit (95% CIs) between the two models within groups defined by M_1_ risk.

*NB* and *DNB* vary widely between groups defined by M_1_ risk (Figure 3). Individuals with intermediate risk (approx. > 5*%* to 25%) receive a significantly higher benefit if screened with M_2_, but individuals with very low ( < 5*%*) or high ( > 25*%*) risk receive negligible benefit if screened with M_2_, because the latter groups are too far from the risk threshold for a new model to change whether they are selected for treatment.

### 6.5. Varying treatment cost, efficacy and threshold

**Figure 4:**
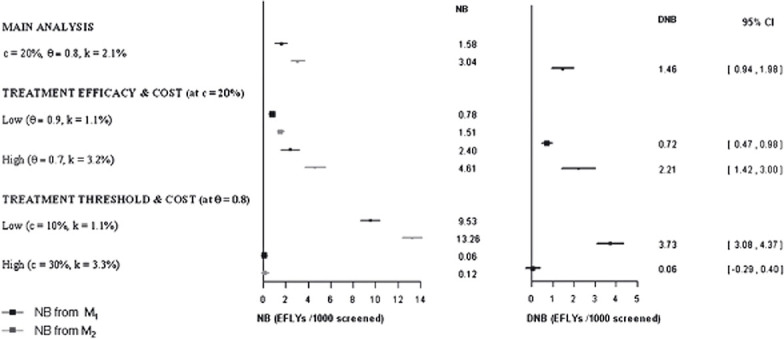
Results from sensitivity analyses on the values used to estimate net benefit. Abbr. c treatment threshold, θtreatment hazard ratio, k relative treatment cost. Units are in EFLYs gained per 1000 screened.

We examined how cost-effectiveness varies with different treatment thresholds, treatment-associated costs and treatment efficacy (Figure 4). Note that we vary pairs of these attributes according to Equation (9) (keeping the third attribute constant) to maintain the rational treatment condition discussed in Section 3.3. At a given treatment threshold, increasing treatment efficacy results in higher net benefits and higher *DNB*, despite the accompanying increase in treatment-related cost. Similarly, for a given treatment efficacy, cheaper interventions offered at low risk thresholds are much more cost-effective than expensive interventions targeted towards high risk individuals. In terms of cost-effectiveness comparisons, the gain from M_2_ appears to be scale-dependent, that is it increases with increasing net benefit from M_1_.

### 6.6. Results from sensitivity analyses and methodology extensions

**Figure 5:**
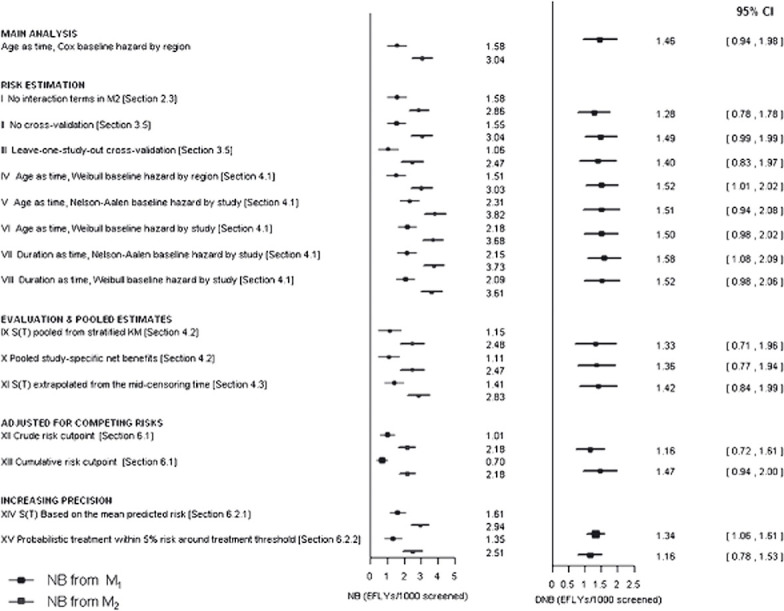
Results from methodology-related sensitivity analyses and extensions (based on the ‘optimal cutpoint’ treatment scenario) with respect to the net benefit (NB) gained using each model compared to no screening/treatment and the difference in net benefit (DNB) gained using M_2_ instead of M_1_. Intermediate estimates are provided in Tables A3 and A4. Units are in EFLYs gained per 1000 screened.

Estimates were robust against a wide range of modelling assumptions (see Figure 5 and supplementary Tables A3, A4 and A5). Different cross-validation approaches, alternative timescales, use of parametric hazards, use of study-specific hazards, different ways to evaluate benefit and different ways to combine study-specific estimates all produced similar results. Notably, precision increased dramatically using predicted risks in the evaluation step (CI widths almost halved) and more modestly using probabilistic treatment. Adjusting for competing risks with the ‘crude risk cutpoint’ reduced *DNB* by about 20% (largely due to lower treatment rates), but with the ‘cumulative risk cutpoint’ *DNB* was similar to the main analysis (Table A5).

## 7. Discussion

### 7.1. Summary

Our method evaluates the clinical benefit from using a prognostic model to inform treatment decisions. It extends recent work on reclassification and decision curves [[12–14] by estimating the cost-benefit of reclassification, taking a fully longitudinal perspective (with appropriate handling of censoring) and broadening the methodology to meta-analysis of individual participant data, an increasingly important resource in epidemiological investigation [27]. Importantly, by quantifying differences in life years the clinical impact of improvements in prognostic accuracy can be formally assessed by extending to full health economic evaluations.

We illustrated our method comparing a model containing just year of birth, age, gender and region versus one containing 3 additional established risk factors. Using model M_1_ to make treatment decisions should not be confused with the argument presented for the ‘poly-pill’ [28], because our estimates ignore differences in screening costs, one of the most important merits of the ‘poly-pill’ approach. Because the risk factors in M_2_ are widely used clinically to predict CVD risk [29–32], it was reassuring to find a clear clinical benefit at 1.46 EFLYs per 1000 screened. The stability of the results across a range of sensitivity analyses also supports the validity of the proposed methods.

To help relate the estimated quantities to their health economics context, consider the following example. Suppose these quantities were obtained from a full economic evaluation, having accounted for detailed costs and benefits. Also, assume that the screening programme was applied to the population of England and Wales, over half of which would be eligible for screening ( ∼ 26.6 million were aged over 40 in 2008). Assuming that the value of one EFLY is £20 000 [Bibr b26] and that the population of England and Wales aged above 40 has the same risk distribution as that in the ERFC data, using M_2_ instead of M_1_ would make a net saving equivalent to 26.6 ×10^6^ ×1.46 ×10^−3^ = 38 836 EFLYs or *£*20 000 ×38 836 = *£*776.2 million in the first 10 years – a substantial saving.

In this discussion, we explain the rationale behind our choice of methodology for the main and sensitivity analyses, highlight important aspects and limitations and suggest possible extensions to refine our approaches. Finally, we consider the role of our methodology in health economic analysis to inform screening policy.

### 7.2. Choice of methodology

The multistudy setting makes it possible to explore heterogeneity between cohorts. We allowed for such heterogeneity when estimating the Cox model coefficients. However, by constructing cross-validation groups by sampling individuals from all cohorts, our main analysis ignored the impact of heterogeneity. Sensitivity analysis III used a preferable procedure, constructing cross-validation groups by sampling cohorts, and showed only a small increase in uncertainty.

In the evaluation step we estimated observed risks by applying the Kaplan–Meier estimator to the subset of individuals across all studies whose risk was above the threshold, thereby treating them as a homogeneous group. The approaches of sensitivity analyses IX, X and X, where observed risks and/or net benefit are estimated within each study before combining, would be more appropriate to handle cases where significant heterogeneity remains among these individuals.

Adjustment for competing risks is not commonly done in work on prognostic models. We have shown that the net benefit comparison changes little when adjusting for competing risks, provided that the risk threshold is lowered to obtain equivalent treated rates to those under crude risks.

Varying treatment cost, efficacy or risk threshold changed net benefits and *DNB* quantitatively, but conclusions about which model is more cost-effective remained the same. At worst, *DNB* became insignificant (with high treatment cost and threshold).

We did not explicitly incorporate ‘false negatives’ into our net benefit estimation, that is, individuals who experienced events within 10 years but their predicted risks were below the treatment threshold. False negatives are relevant to evaluating a strategy of ‘treat all’, which is only of theoretical interest in the risk management of CVD. Our assumed baseline strategy is ‘treat using M_1_’.

### 7.3. Importance of calibration

The net benefit is sensitive to miscalibration, especially for risks close to the treatment threshold. If risk is over-estimated, some individuals who do not benefit will be treated; if risk is under-estimated, some individuals who would benefit from treatment will not be treated. In both cases, net benefit is reduced, provided that models are compared at combinations of *c*, *k* and *θ* that satisfy condition (9). Where condition (9) is not satisfied, miscalibration can lead to spurious cost-effectiveness comparisons, as explained in Section 3.3.

In our data, overall calibration was good (Section 6.2). However, calibration was poor in some cohorts because region-specific (not cohort-specific) risks were used, mimicking the situation when applying a prognostic model to external data. Thus, our analysis allows for miscalibration arising from heterogeneity in risk between cohorts. By comparing the main analysis with sensitivity analyses V to VIII, where cohort-specific baseline hazards gave superior cohort-specific calibration, we conclude that the effect of poor cohort-specific calibration is detrimental to absolute net benefits but has little impact on differences in the net benefit between models with similar (mis)calibration.

### 7.4. Limitations and extensions

Cost-effectiveness comparisons depend on age, gender and other risk factors, so our overall estimates apply to the particular risk profile found in the ERFC data, not the general population. To make results comparable across different datasets and economic evaluations, estimates could be standardised to country-specific risk factor and age distributions.

Our models assume that proportional hazards hold for all risk factors. Where evidence suggests otherwise, models could be modified by stratification or including time-dependent interactions.

Parameter dimensionality is an important aspect of model comparisons. Our approach does not penalise larger models, although cross-validation avoids inappropriately favouring them. However, larger models typically have larger data collection (screening) costs, which would become apparent in a fuller health economic analysis as discussed in Section 7.5.

The hazard ratio of 0.8 for statin efficacy is based on meta-analysis of clinical trials [15]. For this efficacy level to be realistic, we assumed that our modelled population has the same compliance pattern as the clinical trial participants analysed in [15]. Patterns and rates of compliance can easily be incorporated into the analysis, using evidence from relevant compliance studies (e.g. [33]). As described in [34], if 50% of high-risk individuals never even received a prescription, then both cost and benefit would simply halve, and overall conclusions would be little changed. However, if noncompliance took the form of failing to use prescriptions redeemed, treatment benefit would reduce without an equivalent reduction in direct treatment costs.

All our estimates (CVD risk, treatment cost, treatment benefit) are based on a 10-year horizon assuming individuals are screened only once during this time. By modelling hazards using age as the timescale, we can extend the time horizon for prediction to lifetime, as in [35]. Because cost-effectiveness increases with increasing risk, *DNB* estimates that in the short term appear rather small are likely to be magnified over the life-course. Furthermore, we assume that the optimal treatment decisions are based on 10-year risk alone, irrespective of age, sex, and other risk factors. Longer-term risks are also particularly attractive for defining different treatment thresholds for different subgroups. To estimate cost-effectiveness for a subgroup given treatment cost *k* and treatment efficacy *θ* we would first decide on a clinically meaningful time horizon *T*^′^ (e.g. 30 years for a subgroup aged 50–60, 5 years for those over 80) and estimate the new ‘optimal cutpoint’ *c*^′^ for this subgroup using Equation (9). A realistic evaluation of cost-effectiveness over extended periods would also need to consider competing risks and repeated risk assessments. However, currently the long-term cost-effectiveness of prognostic models cannot be formally assessed because of the uncertainty in the long-term benefits and harms of statin treatment. Also, most observational studies lack sufficient follow-up to evaluate long term risks without parametric assumptions. Electronic health records are an increasingly popular data source for pharmaco-epidemiological investigations (e.g. [35]) and have features that could overcome some of these limitations [36].

The proposed framework readily accommodates combination of observational with randomised studies, as we confirmed in a sensitivity analysis (not shown) by including data from untreated arms of six large clinical trials. However, the baseline hazard for each clinical trial needs to be modelled separately, not mixed with that from other studies in the same region, because clinical trials typically include individuals with baseline risks not comparable to the general population.

### 7.5. Towards a full health economic analysis

In our example, comparison of net benefit and comparison of discrimination gave similar qualitative findings. However, our method allows further quantitative comparisons to be made. For example, if the estimated *DNB* was clearly small compared to the additional cost of screening, then we would not prefer model M_2_. To formalise this conclusion we would need a full economic evaluation, which requires a number of further components.

Our model considers only two health states: alive and without a CVD event, and dead or with a CVD event. Further development would allow for impaired quality of life after a first CVD event and for other disease events, as well as for other risk-related treatments, with results expressed in quality-adjusted life years.

A full economic evaluation would consider costs and benefits in more detail. Screening costs would include laboratory tests, healthcare professional time and likely harms (e.g. biopsy and other invasive tests). Treatment costs would include direct costs (e.g. prescription cost, dispensing cost, GP visits and lab tests to monitor for side-effects) and indirect costs (e.g. disutility of receiving treatment, potential for serious side-effects, potential for increasing competing risks, increased frequency of contact with health-care professionals). Benefits would include healthy life years gained from treatment but also societal costs averted such as secondary care costs, disability resulting from first event, and costs to the employer. The specific costs and benefits considered would also depend on the perspective of the evaluation, for example health provider or societal. It would be straightforward to discount costs and benefits over time.

A full evaluation would also allow for differential screening uptake and treatment compliance. Screening approaches based on administrative records (e.g. age, gender, ethnicity, and socioeconomic rank) will clearly capture more people than those requiring prior contact with a healthcare practitioner. Similarly, compliance levels may be influenced by the risk factors measured [37]. For example, healthy individuals may be more compliant with a daily medication that has no obvious health benefits in the short-term if they can monitor changes in their risk factors.

As well as evaluating a strategy of screening everyone, more complex strategies could be considered and evaluated using our methodology. In sequential screening strategies, risk is re-assessed with a more costly model only in individuals initially classified as being within the ‘uncertain’ risk range of a simpler model. Figure 3 suggests that this could be a suitable approach for CVD screening, with model M_2_ used only if model M_1_ risk is within the 10–30% range. Alternatively, in repeated screening strategies, individuals within the ‘uncertain’ risk range of one model could be later assessed either with the same or a more complex model.

### 7.6. Data requirements for effective estimation

Using a data set of 170 000 individuals, the *DNB* was about six times its standard error. This suggests that with a data set nine times smaller (which is still large), sampling error would be of the same order of magnitude as the net benefit of model M_2_ compared with M_1_, making it hard to draw reliable conclusions about a model that is widely believed to be clinically superior. When subtler differences are explored, *DNB* is likely to be smaller. We therefore believe that effective evaluation will require large data sets and probably pooling of data over many cohorts. Multiple cohorts are also needed to give generalizable findings, but heterogeneity between cohorts may increase the imprecision of the overall estimates.

## Appendix A

Membership of the Emerging Risk Factors Collaboration

**Emerging Risk Factors Collaboration Investigators: AFTCAPS**: RW Tipping; **ALLHAT**: CE Ford, LM Simpson; **ARIC**: AR Folsom, LE Chambless; **ATTICA**: DB Panagiotakos, C Pitsavos, C Chrysohoou, C Stefanadis; **BHS**: M Knuiman; **BRHS**: PH Whincup, SG Wannamethee, RW Morris; **BRUN**: S Kiechl, J Willeit, F Oberhollenzer, A Mayr; **BUPA**: N Wald; **BWHHS**: DA Lawlor; **CaPS**: JW Yarnell, J Gallacher; **CASTEL**: E Casiglia, V Tikhonoff; **CHARL**: PJ Nietert, SE Sutherland, DL Bachman, JE Keil; **CHS**: M Cushman, R Tracy, see http://www.chs-nhlbi.org for acknowledgements; **COPEN**: A Tybjærg-Hansen, BG Nordestgaard, R Frikke-Schmidt; **CUORE**: S Giampaoli, L Palmieri, S Panico, D Vanuzzo, L Pilotto; **DRECE**: A Gómez de la Cámara, JA Gómez Gerique; **DUBBO**: L Simons, J McCallum, Y Friedlander; **EAS**: AJ Lee; **EPESEBOS**: J Taylor, JM Guralnik; **EPESEIOW**: R Wallace, JM Guralnik; **EPESENCA**: DG Blazer, JM Guralnik; **EPESENHA**: JM Guralnik; **EPICNOR**: K-T Khaw; **ESTHER**: B Schöttker, H Müller, D Rothenbacher; **FIA**: J-H Jansson, P Wennberg; **FINE_FIN**: A Nissinen; **FINE_IT**: C Donfrancesco, S Giampaoli; **FINRISK92, FINRISK97**: V Salomaa, K Harald, P Jousilahti, E Vartiainen; **FLETCHER**: M Woodward; **FRAMOFF**: RB D'Agostino Sr., PA Wolf, RS Vasan, MJ Pencina; **GLOSTRUP**: E-M Bladbjerg, T Jørgensen, L Møller, J Jespersen; **GOH**: R Dankner, A Chetrit, F Lubin; **GOTO43**: A Rosengren, G Lappas, H Eriksson; **GOTOW**: C Björkelund, L Lissner, C Bengtsson; **GRIPS**: D Nagel; **HISAYAMA**: Y Kiyohara, H Arima, Y Doi, T Ninomiya; **HONOL**: B Rodriguez; **HOORN**: JM Dekker, G Nijpels, CDA Stehouwer; **IKNS**: H Iso, A Kitamura, K Yamagishi, H Noda; **ISRAEL**: U Goldbourt; **KIHD**: J Kauhanen, JT Salonen; **LEADER**: JA Cooper; **MCVDRFP**: WMM Verschuren, A Blokstra; **MESA**: M Cushman, AR Folsom, S Shea, see http://www.mesa-nhlbi.org for acknowledgements; **MOGERAUG1, MOGERAUG2, MOGERAUG3**: A Döring, C Meisinger; **MORGEN**: WMM Verschuren, A Blokstra, HB Bueno-de-Mesquita; **MOSWEGOT**: A Rosengren, G Lappas; **MRFIT**: LH Kuller, G Grandits; **NHANES III**: RF Gillum, M Mussolino; **NPHS II**: JA Cooper, KA Bauer; **NSHS**: S Kirkland, J Shaffer, MR Korin; **OSAKA**: A Kitamura, H Iso, S Sato; **PRIME**: P Amouyel, D Arveiler, A Evans, J Ferrières; **PROCAM**: H Schulte, G Assmann; **PROSPER**: RG Westendorp, BM Buckley, CJ Packard, N Sattar; **QUEBEC**: B Cantin, J-P Després, GR Dagenais; **RANCHO**: E Barrett-Connor, DL Wingard, R Bettencourt; **REYK**: V Gudnason, T Aspelund, G Sigurdsson, B Thorsson; **ROTT**: J Witteman, I Kardys, H Tiemeier, A Hofman; **SHHEC**: H Tunstall-Pedoe, R Tavendale, GDO Lowe, M Woodward; **SHS**: BV Howard, Y Zhang, L Best, J Umans; **SPEED**: Y Ben-Shlomo, G Davey-Smith; **TROMSO**: I Njølstad, T Wilsgaard; **ULSAM**: E Ingelsson, L Lind, V Giedraitis, L Lannfelt; **USPHS**: JM Gaziano, M Stampfer, P Ridker; **USPHS2**: JM Gaziano, P Ridker; **WHI-HaBPS**: S Wassertheil-Smoller, JE Manson; **WHITE I**: M Marmot, R Clarke, A Fletcher; **WHITE II**: E Brunner, M Shipley; **WHS**: P Ridker, J Buring; **WOSCOPS**: J Shepherd, SM Cobbe, I Ford, M Robertson; **ZARAGOZA**: A Marín Ibañez; **ZUTE**: EJM Feskens, D Kromhout. **ERFC website:** http://ceu.phpc.cam.ac.uk/research/erfc/

Data management team: Matthew Walker, Sarah Watson.

**Coordinating Centre:** Myriam Alexander, Adam S Butterworth, Emanuele Di Angelantonio, Oscar H Franco, Pei Gao, Reeta Gobin, Philip Haycock, Stephen Kaptoge, Sreenivasa R Kondapally Seshasai, Sarah Lewington, Lisa Pennells, Eleni Rapsomaniki, Nadeem Sarwar, Alexander Thompson, Simon G Thompson, Matthew Walker, Sarah Watson, Ian R White, Angela M Wood, David Wormser, Xiaohui Zhao, John Danesh (principal investigator).
