# Supplementary material for: A framework for quantifying net benefits of alternative prognostic models
Source: Stat Med. 2011 Sep 9;31(2):114–30. doi: 10.1002/sim.4362 (PMC3496857; doi:10.1002/sim.4362)
Supplement: Supplementary file 1 [file sim0031-0114-SD1.doc]

Web appendix

Table A1. Characteristics of individual studies contributing to the analysis.

| **Region** | **Study abbrev.** | **No. of participants** | **Mean (SD) age at survey** | **% Male** | **% Current smokers** | **Mean (SD) Systolic Blood Pressure** | **Mean (SD) Total Cholesterol** | **Mean (SD) follow-up (years)** | **Time to mid-censoring[[1]](#footnote-2)** | **Number (%) CVD events** |
| --- | --- | --- | --- | --- | --- | --- | --- | --- | --- | --- |
| N. EUROPE | BRHS | 6345 | 50.1(5.7) | 100% | 40.9% | 144.7(20.7) | 6.3(1.0) | 19.2(6.3) | 23.1 | 1501 (23.7%) |
|  | BWHHS | 2927 | 68.5(5.5) | 0% | 11.3% | 146.9(25.0) | 6.7(1.2) | 4.3(1.1) | 4.6 | 86 (2.9%) |
|  | CAPS | 2077 | 52.1(4.6) | 100% | 54.9% | 140.9(19.3) | 5.7(1.1) | 11.7(2.9) | 13.0 | 256 (12.3%) |
|  | COPEN | 7370 | 60.9(11.9) | 42% | 48.0% | 140.1(22.4) | 6.2(1.3) | 6.3(2.4) | 7.5 | 503 (6.8%) |
|  | EAS | 958 | 64.2(5.6) | 50% | 23.1% | 140.8(22.0) | 6.9(1.3) | 12.1(4.6) | 15.3 | 125 (13.0%) |
|  | ESTHER | 4138 | 61.3(6.5) | 42% | 14.2% | 139.2(19.7) | 5.8(1.3) | 2.1(0.4) | 2.0 | 63 (1.5%) |
|  | FINE_FIN | 248 | 76.4(4.8) | 100% | 13.3% | 156.2(21.9) | 5.7(1.1) | 6.2(3.3) | 10.0 | 90 (36.3%) |
|  | FINRISK92 | 3494 | 51.7(7.0) | 46% | 26.2% | 140.7(20.2) | 5.9(1.1) | 11.2(2.1) | 11.9 | 235 (6.7%) |
|  | FINRISK97 | 4993 | 54.5(9.0) | 50% | 24.3% | 140.0(20.2) | 5.8(1.0) | 6.6(0.9) | 6.8 | 171 (3.4%) |
|  | GOTO43 | 723 | 50.0 (0.0) | 100% | 30.7% | 129.7(16.0) | 5.8(1.0) | 9.8(1.4) | 10.0 | 32 (4.4%) |
|  | GOTOW | 695 | 70.0 (5.8) | 0% | 20.6% | 154.4(23.5) | 6.4(1.1) | 7.6(1.8) | 8.2 | 85 (12.2%) |
|  | GRIPS | 5670 | 47.7(5.1) | 100% | 37.6% | 131.8(15.8) | 5.6(1.0) | 9.3(1.7) | 9.8 | 382 (6.7%) |
|  | HOORN | 1997 | 61.0 (7.3) | 44% | 32.5% | 133.7(19.7) | 6.7(1.2) | 8.3(1.8) | 8.9 | 102 (5.1%) |
|  | KIHD | 1969 | 52.4(5.3) | 100% | 30.6% | 130.5(17.0) | 5.9(1.0) | 16.9(6.2) | 20.9 | 502 (25.5%) |
|  | MOGERAUG1 | 836 | 53.8(5.8) | 100% | 33.6% | 136.7(17.8) | 6.3(1.2) | 11.7(3.0) | 13.0 | 74 (8.9%) |
|  | MOGERAUG2 | 1510 | 56.1(9.7) | 100% | 27.2% | 137.8(18.3) | 6.4(1.2) | 7.0(2.1) | 7.9 | 74 (4.9%) |
|  | MOSWEGOT | 2745 | 52.2(7.2) | 46% | 28.5% | 131.4(18.8) | 6.1(1.2) | 12.0(4.3) | 13.0 | 218 (7.9%) |
|  | NPHSII | 1835 | 61.4(3.5) | 100% | 19.7% | 134.3(17.9) | 5.6(1.0) | 3.3(1.4) | 3.5 | 91 (5.0%) |
|  | PRIME | 9210 | 54.8(2.9) | 100% | 26.7% | 133.3(18.6) | 5.7(1.0) | 5.5(1.0) | 5.2 | 172 (1.9%) |
|  | PROCAM | 14239 | 48.8(6.1) | 74% | 34.7% | 131.9(18.6) | 5.9(1.1) | 10.4(5.5) | 9.0 | 512 (3.6%) |
|  | REYK | 1415 | 61.7(7.3) | 24% | 32.4% | 137.0(20.8) | 6.6(1.2) | 14.8(4.7) | 17.7 | 196 (13.9%) |
|  | ROTT | 4044 | 67.3(8.1) | 40% | 19.6% | 137.8(21.8) | 6.7(1.2) | 10.9(3.3) | 12.5 | 304 (7.5%) |
|  | SHHEC | 8450 | 50.4(6.1) | 51% | 44.2% | 132.1(19.1) | 6.4(1.2) | 9.6(1.6) | 10.0 | 375 (4.4%) |
|  | SPEED | 2001 | 54.7(4.4) | 100% | 47.5% | 139.8(23.1) | 5.9(1.2) | 14.7(4.6) | 17.2 | 300 (15.0%) |
|  | TROMSO | 7865 | 47.7(5.8) | 54% | 43.0% | 132.0(17.0) | 6.3(1.2) | 13.2(2.6) | 14.0 | 567 (7.2%) |
|  | ULSAM | 1678 | 54.1(8.6) | 100% | 55.6% | 135.1(18.6) | 6.8(1.4) | 21.3(10.5) | 34.1 | 569 (33.9%) |
|  | WHITEII | 7982 | 49.0 (5.5) | 69% | 20.8% | 120.7(13.8) | 6.4(1.2) | 8.0(2.2) | 7.8 | 196 (2.5%) |
|  | ZUTE | 360 | 75.4(4.4) | 100% | 31.9% | 150.1(20.8) | 6.1(1.1) | 7.0(3.4) | 10.0 | 86 (23.9%) |
| S. EUROPE | ATENA | 4261 | 50.7(6.7) | 0% | 39.6% | 134.6(21.0) | 6.2(1.2) | 6.7(0.9) | 6.8 | 22 (0.5%) |
|  | BRUN | 790 | 57.5(11.3) | 49% | 24.7% | 144.4(21.1) | 5.7(1.0) | 13.3(4.0) | 15.5 | 83 (10.5%) |
|  | FINE_IT | 432 | 72.5(4.5) | 100% | 27.5% | 166.6(22.0) | 5.8(1.1) | 10.7(6.6) | 21.4 | 149 (34.5%) |
|  | MATISS83 | 1082 | 53.2(8.4) | 100% | 50.7% | 139.6(21.2) | 5.9(1.2) | 15.6(4.9) | 18.7 | 94 (8.7%) |
|  | MATISS87 | 1873 | 53.5(8.2) | 45% | 21.4% | 142.1(22.1) | 5.7(1.0) | 13.9(3.5) | 15.6 | 81 (4.3%) |
|  | MATISS93 | 460 | 52.3(7.7) | 100% | 39.6% | 140.8(21.4) | 5.6(1.1) | 8.3(1.6) | 9.2 | 16 (3.5%) |
|  | MONFRI89 | 433 | 50.6(6.9) | 100% | 32.6% | 142.3(20.7) | 6.0(1.3) | 12.4(3.0) | 13.6 | 22 (5.1%) |
|  | ZARAGOZA | 1915 | 60.3(10.7) | 44% | 16.2% | 134.1(15.1) | 6.0(1.0) | 4.9(0.7) | 5.0 | 67 (3.5%) |
| USA & CANADA | ARIC | 12969 | 54.3(5.7) | 43% | 25.9% | 120.0(18.1) | 5.5(1.1) | 13.3(2.9) | 14.3 | 1056 (8.1%) |
|  | CHS1 | 3370 | 72.3(5.2) | 37% | 11.8% | 134.8(21.0) | 5.5(1.0) | 9.5(3.9) | 12.5 | 766 (22.7%) |
|  | CHS2 | 359 | 72.2(5.2) | 38% | 17.3% | 141.4(22.6) | 5.4(1.0) | 7.5(2.7) | 9.2 | 62 (17.3%) |
|  | EPESEBOS | 416 | 77.2(4.3) | 0% | 11.8% | 136.7(17.2) | 5.8(1.0) | 3.8(0.9) | 4.1 | 30 (7.2%) |
|  | EPESEIOW | 1052 | 77.9(4.7) | 28% | 4.8% | 139.0(17.6) | 5.7(1.1) | 4.3(1.1) | 4.8 | 95 (9.0%) |
|  | EPESENCA | 818 | 77.3(4.7) | 32% | 12.8% | 144.6(21.9) | 5.5(1.1) | 3.7(1.0) | 4.1 | 74 (9.0%) |
|  | EPESENHA | 509 | 77.9(4.8) | 38% | 16.1% | 138.6(18.3) | 5.7(1.1) | 4.0(1.0) | 4.4 | 33 (6.5%) |
|  | FRAMOFF | 2412 | 60.2(9.2) | 43% | 0.0% | 125.5(18.2) | 5.2(0.9) | 5.3(1.3) | 5.3 | 53 (2.2%) |
|  | HONOL | 1820 | 77.6(4.1) | 100% | 8.1% | 148.7(22.3) | 5.0(0.8) | 5.8(1.8) | 6.6 | 192 (10.5%) |
|  | MESA | 5905 | 61.8(10.3) | 46% | 14.8% | 125.4(21.3) | 5.0(0.9) | 4.6(0.8) | 4.8 | 128 (2.2%) |
|  | NSHS | 1070 | 58.8(12.7) | 48% | 22.9% | 130.0(17.9) | 5.7(1.0) | 8.4(2.7) | 9.7 | 43 (4.0%) |
|  | QUEBEC | 1213 | 56.9(7.1) | 100% | 40.0% | 131.2(17.7) | 5.7(1.0) | 5.0(0.8) | 5.3 | 33 (2.7%) |
|  | RANCHO | 1698 | 69(10.2) | 41% | 13.8% | 137.2(21.6) | 5.7(1.0) | 12.2(5.4) | 16.9 | 385 (22.7%) |
|  | SHS | 2074 | 55.5(8.1) | 43% | 40.2% | 124.4(18.3) | 5.0(1.0) | 11.2(3.6) | 13.2 | 246 (11.9%) |
| JAPAN | HISAYAMA | 2372 | 58.7(11.6) | 41% | 12.9% | 132.7(21.1) | 5.3(1.1) | 12.6(3.4) | 14.2 | 258 (10.9%) |
|  | IKNS | 4184 | 57(9.9) | 46% | 25.3% | 133.8(19.0) | 5.1(0.9) | 10.9(2.6) | 12.1 | 118 (2.8%) |
|  | OSAKA | 9914 | 53.6(8.8) | 66% | 37.7% | 124.3(18.0) | 5.3(0.9) | 4.9(2.2) | 5.1 | 85 (0.9%) |
| **TOTAL (53 studies)** | | **171175** | **55.7(10.2)** | **60%** | **29.9%** | **133(20.7)** | **5.9(1.2)** | **9.4(5.2)** | **10.3(5.3)** | **12058 (7.0%)** |

Table A2. Numerical example to show how to estimate the difference in net benefit in a hypothetical study of 5000 subjects using two hypothetical models M_1_ and M_2_. Reference values used for treatment threshold c=20%, treatment efficacy *θ* =0.8 and relative treatment cost k =2.13%[[2]](#footnote-3). The ‘optimal cutpoint’ scenario is applied.

| Prediction models | | M_1_ | M_2_ | |
| --- | --- | --- | --- | --- |
| N screened=5000 subjects | | | | |
| Number to treat  (predicted risk >*c*) | $n_{1}=200$ | | | $n_{2}=250$ |
| Probability of being treated among those screened | $P_{1}=\frac{n_{1}}{N}=0.04$ | | | $P_{2}=\frac{n_{2}}{N}=0.05$ |
| Observed (KM) 10-year survival in the treated | $S_{1}(t=10)=0.75$ | | | $S_{2}(t=10)=0.7$ |
| Benefit (EFLYs) over *T=*10 years per person treated  (in EFLYs)[[3]](#footnote-4) | $\begin{matrix} B_{_{1}}(T)=\int_{0}^{T} \left(S_{1}^{\theta}(u)-S_{1}(u)\right)du \\ \approx\frac{1}{2}T\left(S_{1}^{\theta}(T)-S_{1}(T)\right) \end{matrix}$ = ½*10*(0.75^0.8^-0.75)  =0.222 | | | $\begin{matrix} B_{_{2}}(T)=\int_{0}^{T} \left(S_{2}^{\theta}(u)-S_{2}(u)\right)du \\ \approx\frac{1}{2}T\left(S_{2}^{\theta}(T)-S_{2}(T)\right) \end{matrix}$ =½*10*( 0.7^0.8^-0.7)  =0.259 |
| Treatment cost over 10 years per person treated (in EFLYs)Error: Reference source not found | $\begin{matrix} C_{_{1}}(T)=k\int_{0}^{T} \left(S_{1}^{\theta}(u)\right)du \\ \approx k\frac{1}{2}T\left(S_{1}^{\theta}(T)+1\right) \end{matrix}$ = 0.0213*½*10*(0.75^0.8^+1)  =0.191 | | | $\begin{matrix} C_{_{2}}(T)=k\int_{0}^{T} \left(S_{2}^{\theta}(u)\right)du \\ \approx k\frac{1}{2}T\left(S_{2}^{\theta}(T)+1\right) \end{matrix}$ = 0.0213*½*10*(0.7^0.8^+1)  =0.187 |
| Net benefit per person screened (in EFLYs) | $NB_{1}(T)=P_{1}\left[B_{_{1}}(T)-C_{_{1}}(T)\right]$ =0.04*(0.222-0.191) =0.00124  or 1.24 EFLYs per 1000 persons screened | | | $NB_{2}(T)=P_{2}\left[B_{_{2}}(T)-C_{_{2}}(T)\right]$ =0.05*(0.259-0.187) =0.0036  or 3.6 EFLYs per 1000 persons screened |
| *Difference* in net benefit per person screened (in EFLYs) | $DNB=NB_{2}-NB_{1}$=3.6-1.24=2.36 EFLYs (per 1000 screened) | | | |
| Incremental cost-effectiveness ratio (ICER) | $ICER=\frac{P_{2}C_{_{2}}-P_{1}C_{_{1}}}{P_{2}B_{_{2}}-P_{1}B_{_{1}}}=\frac{0.05*0.187-0.04*0.191}{0.05*0.259-0.04*0.222}=0.42$ | | | |

Table A3. Detailed estimates from sensitivity analyses and methodological extensions (risk estimation).

| TITLE | **Section** | **Abbr** | **C-index[[4]](#footnote-5)** | | **Calibration[[5]](#footnote-6)**  *χ^2^* **(P-value)** | | **treated per 1000** | | **1-S(t=10)** | | **Benefit** | | **Cost** | | **Net Benefit (SE)** | | **DNB (SE)** |
| --- | --- | --- | --- | --- | --- | --- | --- | --- | --- | --- | --- | --- | --- | --- | --- | --- | --- |
|  |  |  |  |  |  |  |  |  |  |  | **all in EFLYs[[6]](#footnote-7)  per 1000 screened** | | | | | | |
|  |  |  | **M1** | **M2** | **M1** | **M2** | **M1** | **M2** | **M1** | **M2** | **M1** | **M2** | **M1** | **M2** | **M1** | **M2** |  |
| MAIN ANALYSIS | main | | 0.686 | 0.736 | 17.4 (0.236) | 32.9 (0.003) | 51.6 | 58.3 | 0.265 | 0.282 | 11.51 | 14.14 | 9.92 | 11.10 | 1.58 (0.28) | 3.04 (0.32) | **1.46 (0.26)** |
| No interaction terms in M_2_ | 2.3 | I | 0.686 | 0.735 | 17.4 (0.236) | 66.7 (0) | 51.6 | 58.0 | 0.265 | 0.280 | 11.51 | 13.91 | 9.92 | 11.05 | 1.58 (0.28) | 2.86 (0.31) | **1.28 (0.25)** |
| No cross-validation | 3.5 | II | 0.687 | 0.737 | 17.3 (0.243) | 30.5 (0.007) | 52.5 | 59.0 | 0.264 | 0.281 | 11.66 | 14.26 | 10.11 | 11.23 | 1.55 (0.28) | 3.04 (0.32) | **1.49 (0.25)** |
| Leave one study out cross-validation | 3.5 | III | 0.680 | 0.731 | 31 (0.006) | 41.7 (0) | 52.2 | 59.6 | 0.250 | 0.269 | 11.15 | 13.88 | 10.09 | 11.42 | 1.06 (0.3) | 2.47 (0.35) | **1.40 (0.29)** |
| Age timescale, Weibull baseline hazard by region | 4.1 | IV | 0.687 | 0.736 | 11.9 (0.617) | 27.6 (0.016) | 55.7 | 60.2 | 0.254 | 0.278 | 12.24 | 14.49 | 10.73 | 11.47 | 1.51 (0.29) | 3.03 (0.33) | **1.52 (0.26)** |
| Age timescale, Nelson-Aalen baseline hazard by study | 4.1 | V | 0.635 | 0.692 | 10.1 (0.752) | 24.3 (0.042) | 51.3 | 59.2 | 0.282 | 0.295 | 12.11 | 15.01 | 9.80 | 11.19 | 2.31 (0.28) | 3.82 (0.32) | **1.51 (0.28)** |
| Age timescale, Weibull baseline hazard by study | 4.1 | VI | 0.627 | 0.688 | 7.1 (0.931) | 27.7 (0.016) | 55.6 | 64.2 | 0.274 | 0.286 | 12.84 | 15.86 | 10.65 | 12.18 | 2.18 (0.29) | 3.68 (0.32) | **1.50 (0.27)** |
| Duration timescale, Nelson-Aalen baseline hazard by study | 4.1 | VII | 0.629 | 0.689 | 28.9 (0.011) | 21.2 (0.098) | 50.5 | 60.6 | 0.270 | 0.284 | 11.81 | 15.23 | 9.66 | 11.49 | 2.15 (0.29) | 3.73 (0.32) | **1.58 (0.26)** |
| Duration timescale, Weibull baseline hazard by study | 4.1 | VIII | 0.629 | 0.689 | 20.6 (0.112) | 29.3 (0.01) | 54.8 | 65.5 | 0.269 | 0.277 | 12.60 | 16.06 | 10.51 | 12.45 | 2.09 (0.27) | 3.61 (0.32) | **1.52 (0.27)** |
| Adjusted for competing risks ("crude risk cutpoint") | 6.1 | XII | 0.687 | 0.737 | 13.8 (0.462) | 31.4 (0.005) | 40.8 | 50.2 | 0.241 | 0.258 | 8.29 | 11.19 | 7.28 | 9.01 | 1.01 (0.24) | 2.18 (0.27) | **1.16 (0.23)** |
| Adjusted for competing risks ("cumulative risk cutpoint") | 6.1 | XIII | 0.787 | 0.737 | 13.8 (0.462) | 31.4 (0.005) | 51.8 | 58.5 | 0.227 | 0.250 | 10.06 | 12.74 | 9.35 | 10.56 | 0.7 (0.25) | 2.18 (0.30) | **1.47 (0.26)** |
| Adjusted for competing risks ("cumulative risk cutpoint") | XIII | XIII | 0.721 | 0.761 | 13.8 (p=0.462) | 31.4 (p=0.005) | 51.8 | 58.5 | 0.227 | 0.250 | 10.06 | 12.74 | 9.35 | 10.56 | 0.7 (0.25) | 2.18 (0.30) | **1.47 (0.26)** |

Table A4. Detailed estimates from sensitivity analyses and methodological extensions (risk evaluation).

| TITLE | **Section** | **Abbr.** | **Treated per 1000** | | **1-S(t=10)** | | **Benefit** | | **Cost** | | **Net Benefit (SE)** | | **DNB (SE)** |
| --- | --- | --- | --- | --- | --- | --- | --- | --- | --- | --- | --- | --- | --- |
|  |  |  |  |  |  |  | **all in EFLYs[[7]](#footnote-8)  per 1000 screened** | | | | | | |
|  |  |  | **M1** | **M2** | **M1** | **M2** | **M1** | **M2** | **M1** | **M2** | **M1** | **M2** |  |
| MAIN ANALYSIS | main | | 51.6 | 58.3 | 0.265 | 0.282 | 11.51 | 14.14 | 9.92 | 11.10 | 1.58 (0.28) | 3.04 (0.32) | **1.46 (0.26)** |
| Stratified KM | 4.2 | IX | 51.6 | 58.3 | 0.236 | 0.259 | 11.12 | 13.63 | 9.97 | 11.15 | 1.15 (0.33) | 2.48 (0.37) | **1.33 (0.32)** |
| Pooled study-specific estimates | 4.3 | X | 51.6 | 58.3 | 0.265 | 0.282 | 10.97 | 13.59 | 9.86 | 11.12 | 1.11 (0.31) | 2.47 (0.37) | **1.36 (0.30)** |
| Extrapolation from mid-censoring time | 4.3 | XI | 51.6 | 58.3 | 0.252 | 0.269 | 11.35 | 13.95 | 9.94 | 11.12 | 1.41 (0.29) | 2.83 (0.34) | **1.42 (0.29)** |
| Based on the mean predicted risk | 6.2.1 | XIV | 51.6 | 58.3 | 0.249 | 0.271 | 11.53 | 14.05 | 9.92 | 11.11 | 1.61 (0.23) | 2.94 (0.27) | **1.34 (0.14)** |
| Probabilistic treatment within 5% around treatment threshold | 6.2.2 | XV | 53.7 | 62.2 | 0.258 | 0.268 | 11.72 | 14.42 | 10.37 | 11.91 | 1.35 (0.25) | 2.51 (0.29) | **1.16 (0.19)** |

Table A5. Cost-effectiveness estimates based on the 10-year cumulative CVD incidence adjusting for competing causes of death. Two types of treatment threshold are presented. Under the “crude risk cutpoint” individuals are treated according to the treatment threshold used for crude risks (*c*=20%). The “cumulative incidence cutpoint” is the cutpoint applied to cumulative risks so that each model treats the same number of individuals as under crude risks at *c*=20%. Because cumulative risks are lower than crude risks treatment thresholds are now lower.

| **Estimate** | **"Crude risk cutpoint"** | | | **“Cumulative-incidence cutpoint”** | | |
| --- | --- | --- | --- | --- | --- | --- |
|  | **M_1_** | **M_2_** | ***Difference (SE)*** | ***M_1_*** | **M_2_** | ***Difference (SE)*** |
| Treatment threshold for 10-year risk (c) | 20% | 20% | *fixed by method* | *18.4%* | 18.8% | *0.4%* |
| Number treated per 1000 screened (P x 1000) | 40.8 | 50.2 | *9.4* | *51.8* | 58.5 | *6.7* |
| 10-year cumulative risk in treated [$1-I^{CR}(T)$] | 24.1% | 25.8% | *1.7%* | 22.7% | 25.0% | *2.4%* |
| **Difference in Net Benefit**  **(DNB x 1000)** | **1.01** | **2.18** | ***1.16 (0.23)*** | **0.70** | **2.18** | ***1.47 (0.26)*** |

1. when 50% of the individuals in the study have been censored. [↑](#footnote-ref-2)
2. Value of *k* is computed from *c* and *θ* using equation (11). [↑](#footnote-ref-3)
3. To simplify the calculations shown in this example the trapezium rule is applied once across the whole 10-year time interval.

   EFLY: event-free life year [↑](#footnote-ref-4)
4. overall estimate is the mean of C-indices computed within each region and gender (or study and gender depending on model stratification) and weighted by the corresponding number of events. [↑](#footnote-ref-5)
5. based on model-specific risk groups formed by splitting the data into 15 equally sized subsets. [↑](#footnote-ref-6)
6.  EFLY: event-free life year; DNB: difference in net benefit; SE standard error from 200 bootstrap samples [↑](#footnote-ref-7)
7.  EFLY: event-free life year; DNB: difference in net benefit; SE standard error from 200 bootstrap samples [↑](#footnote-ref-8)
